# Potential Therapeutic Strategies for Steatosis, Oxidative Stress, Inflammation, and Fibrosis in Liver Disease

**DOI:** 10.3390/ijms27062546

**Published:** 2026-03-10

**Authors:** Pablo Muriel, Eduardo E. Vargas-Pozada, Linda Vanessa Márquez-Quiroga, Erika Ramos-Tovar

**Affiliations:** 1 Departamento de Farmacología, CINVESTAV, Av. Instituto Politécnico Nacional 2508, Col. San Pedro Zacatenco, Alcaldía Gustavo A. Madero, Ciudad de México 07360, Mexico; 2 Sección de Estudios de Posgrado e Investigación, Escuela Superior de Medicina-Instituto Politécnico Nacional, Plan de San Luis Esq. Díaz Mirón s/n, Casco de Santo Tomás, Miguel Hidalgo, Ciudad de México 11340, Mexico

**Keywords:** ursodeoxycholic acid, pirfenidone, S-adenosyl-L-methionine, N-acetylcysteine, liver fibrosis

## Abstract

Liver disease encompasses a wide range of conditions, each requiring tailored therapeutic approaches. This review describes and critically discusses treatments with robust evidence for improving liver health. Ursodeoxycholic acid (UDCA) is a drug approved by the Food and Drug Administration of the USA to treat primary biliary cholangitis (PBC). In addition, UDCA has been demonstrated to protect against metabolic dysfunction-associated steatohepatitis, fibrosis, and drug-induced liver injury (DILI). The mechanism of action of UDCA has been attributed not only to decreasing the effects of toxic bile acids but also to protecting mitochondrial integrity and function, as well as to antioxidant, anti-inflammatory, and anti-apoptotic activities. UDCA can scavenge reactive oxygen species (ROS) and activate the nuclear factor-E2-related factor-2 (Nrf2) pathway, thereby exerting antioxidant activity. The anti-inflammatory activity of UDCA is associated with its ability to inhibit the nuclear factor-κB pathway. Pirfenidone is a well-recognized antifibrotic drug for the treatment of idiopathic pulmonary fibrosis; its effects on liver fibrosis have also been demonstrated. Pirfenidone exerts anti-inflammatory effects by attenuating the nucleotide-binding oligomerization domain-like receptor 3 inflammasome signaling pathway. The antioxidant actions of pirfenidone are associated with its ability to upregulate the Nrf2 pathway. Both the anti-inflammatory and antioxidant properties of pirfenidone act together to attenuate lung and liver fibrosis, decreasing transforming growth factor-β levels, inhibiting profibrogenic hepatic stellate cell activation, and increasing extracellular matrix degradation. Methyltransferases utilize S-adenosyl-L-methionine (SAM) as a methyl donor for most transmethylation reactions in the body. SAM increases reduced glutathione (GSH) levels, exerting important antioxidant effects. Evidence indicates that SAM prevents fibrosis and attenuates hepatocellular carcinoma development, improving patient survival. N-acetylcysteine (NAC) is a precursor to L-cysteine and GSH and is used in clinical settings to treat cancer, nephropathy, heart disease, pulmonary fibrosis, polycystic ovary syndrome, and influenza. Regarding the liver, NAC is the most accepted treatment for DILI, especially after paracetamol overdose. Owing to its antioxidant and anti-inflammatory actions, NAC has been successfully used to treat chronic liver injuries, including hepatosteatosis and fibrosis. Therefore, ursodeoxycholic acid, pirfenidone, S-adenosyl-L-methionine, and N-acetylcysteine could represent therapeutic strategies for the treatment of liver pathologies.

## 1. Introduction

Hepatic steatosis, inflammation, and oxidative stress are interrelated pathological processes in metabolic dysfunction-associated steatotic liver disease (MASLD). Steatosis triggers metabolic stress, leading to increased production of reactive oxygen species (ROS) and oxidative damage. This, in turn, triggers liver damage and immune responses that lead to chronic liver inflammation. The synergistic interaction of these mechanisms underlies the progression from simple fatty liver to steatohepatitis, fibrosis, and cirrhosis. The metabolically dysfunction-associated steatohepatitis (MASH), a more severe form of MASLD, increases the risk of further complications, including cirrhosis and liver cancers [1,2,3]. On the other hand, drug-induced liver injury is a major cause of acute liver failure, with a mortality rate of 10% to 50%. Its etiology includes medications, herbal products, and dietary supplements. Intrinsic, dose-dependent, and predictable liver injury (DILI) is primarily due to substances such as acetaminophen, which causes liver toxicity through direct metabolic pathways [4]. Oxidative stress, mitochondrial dysfunction, and inhibition of bile salt export are some of the pathophysiological factors associated with DILI [5]. Another liver condition is primary biliary cholangitis (PBC), an autoimmune disorder characterized by the progressive destruction of the liver’s small bile ducts [6]. This damage leads to cholestasis and fibrosis and, if left untreated, can progress to cirrhosis and liver failure, making it a critical area for therapeutic intervention and the prevention of liver morbidity.

## 2. Ursodeoxycholic Acid

Ursodeoxycholic acid (3α,7β-dihydroxy-5β-colonic acid; UDCA) was first obtained from polar bear bile and has been demonstrated to help treat gallbladder disorders [7,8,9]. UDCA is the only drug approved by the U.S. Food and Drug Administration (FDA) for the treatment of PBC [6]. UDCA is a natural bile acid produced by the human body that is capable of dissolving gallstones [10,11] and is useful for the treatment of cholestasis [11,12,13,14,15,16,17], biliary pancreatitis [18,19,20], PBC [21,22,23,24,25,26], MASH [27,28,29], fibrosis [30,31,32], and drug-induced liver injury (DILI) [5]. Moreover, UDCA is beneficial for liver transplantation patients [17,33] and improves colon inflammation by promoting macrophage M2 polarization, an anti-inflammatory phenotype [34]. Additionally, UDCA modulates oncogenic signaling pathways, improving patient cancer outcomes [35,36] by increasing apoptosis, attenuating cancer cell growth [37,38], and triggering autophagy [39,40]. Research indicates that UDCA ameliorates mitochondrial dysfunction and has neuroprotective properties [41,42,43,44,45,46,47]. Currently, UDCA can be produced chemically or by biosynthetic mechanisms [48].

### 2.1. Metabolic Dysfunction-Associated Steatohepatitis

Clinical evidence suggests that UDCA can be useful in treating metabolic dysfunction-associated steatotic liver disease (MASLD) by attenuating hepatosteatosis, liver injury, and fibrosis [49,50,51,52,53,54]. Fundamental studies in experimental animals and patients with MASH have shown that UDCA inhibits the miR-34a/SIRT1/p53 signaling pathway to reduce liver steatosis, apoptosis, and inflammation [53]. In addition, UDCA has been reported to protect the liver by limiting the toxicity of bile acids in the small intestine [55]. Evidence indicates that UDCA attenuates hepatosteatosis and insulin resistance by inducing the excretion of hepatic lipids in KK-Ay mice fed a high-fat diet [56]. A meta-analysis revealed that UDCA is useful for attenuating serum markers of liver damage and cholestasis in patients with MASLD [57]. A study investigated the impact of long-term UDCA therapy on circulating levels of the microRNAs miR-34a and miR-506 [58]. The results showed that baseline levels of miR-34a and miR-506 were significantly elevated in PBC patients compared with controls and were significantly reduced after UDCA therapy in PBC, but not in PSC [59]. However, another systematic review indicated that UDCA did not ameliorate the anthropometric and histopathological characteristics in patients with MASH, although the serum markers of liver damage showed some improvement [60].

**Figure 1:**
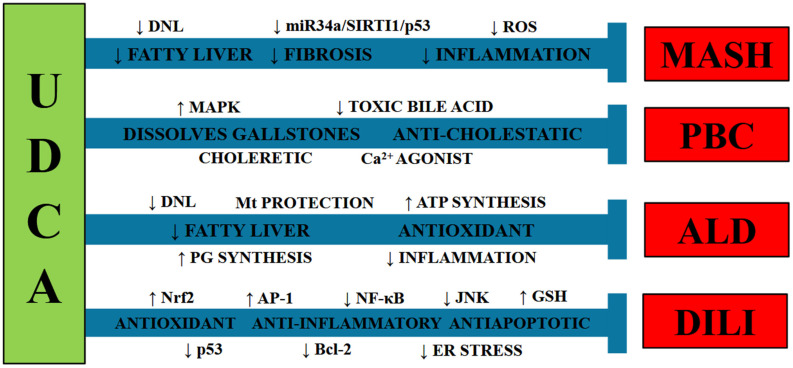
Ursodeoxycholic acid (UDCA) has several beneficial effects on major liver pathologies. UDCA attenuates metabolic dysfunction-associated steatohepatitis (MASH) by reducing fatty liver, inflammation, and fibrosis. UDCA effectively improves primary biliary cholangitis (PBC) by dissolving gallstones and producing choleretic effects. The antioxidant and antisteatotic effects of UDCA protect against alcoholic liver disease (ALD). The antioxidant, anti-inflammatory, and anti-apoptotic effects of UDCA mitigate drug-induced liver disease (DILI). Abbreviations: AP-1: activator protein-1; GSH: reduced glutathione; Mt: mitochondria; DNL: de novo lipogenesis; ROS: reactive oxygen species; PG: prostaglandin; Nrf2: nuclear factor-E2-related factor-2; activator protein-1 (AP-1); NF-κB: nuclear factor-κB; JNK: janus kinases; BCL-2: B-cell lymphoma-2; ER: endoplasmic reticulum.

An interesting study revealed that 24-nor-ursodeoxycholic acid (norUDCA), a UDCA derivative, inhibited de novo lipogenesis (DNL) and apoptosis in a MASH mouse model, attenuating hepatosteatosis [61]. Moreover, norUDCA has been proposed to treat cholestasis in patients with MASLD [62]. A double-masked, randomized, placebo-controlled trial demonstrated the effectiveness of norUDCA in significantly decreasing serum markers of liver damage in patients with MASH [63]. NorUDCA administration to animals fed a Western diet to induce MASH reduced hepatosteatosis, inflammation, and fibrosis in the livers of the treated animals [64]. A combination of UDCA and BAR502, a farnesoid X receptor (FXR) and GPBAR agonist, yielded a greater effect than any drug alone in protecting rodents from MASH induced by a Western diet, reducing the degree of hepatosteatosis, hepatocyte ballooning, hepatic inflammation, and scar tissue deposition [65]. Recently, the FDA approved new drugs for the treatment of MASLD and obesity, closely related diseases. One of the molecular mechanisms of resmeritom, a drug recently approved by the FDA for the treatment of MASLD, is the stimulation of mitochondrial β-oxidation; the molecular target is similar to that of UDCA [66]. However, this selective thyroid hormone receptor beta agonist (THR-β) exerts its effects through heterodimerization of the fatty acid retinoid receptor X [67,68], while UCDA acts on the lipid pathway of the hepatic peroxisome proliferator-activated receptor α (PPAR-α) and cytochrome P450 family 4 subfamily A member 14 (CYP4A14). Promoting lipophagy via two pathways could have synergistic effects on β-oxidation [68]. Moreover, studies have shown that resmeritom reduces miR-34a-mediated suppression of THR-β, and UCDA shows a similar effect; therefore, studies combining these drugs would help elucidate whether they have biological mechanisms that enhance their combined impact. Glucagon-like peptide-1 receptor agonists (GLP-1 RAs), which include semaglutide, tirzepatide, and liraglutide, are also FDA-approved and improve glycemic control, promote weight loss, and reduce systemic inflammation [69]. GLP-1 RAs show significant promise in the treatment of MASLD, particularly in patients with type 2diabetes and obesity [70]. The combination of UCDA, which promotes β-oxidation, and GLP-1 RAs, which regulate glucose by mimicking GLP-1 effects, may benefit both hepatic and extrahepatic manifestations of metabolic disease. However, clinical evidence of the utility of UDCA in patients with MASH is insufficient to recommend this bile acid for treating MASLD/MASH [71] (Figure 1).

### 2.2. Alcoholic Liver Disease

UDCA and its conjugated form, tauroursodeoxycholic acid (TUDCA), prevent ethanol-induced injury in HepG2 cells [66]. Accordingly, UDCA and TUDCA attenuated ethanol-induced damage in human SK-Hep-1 hepatoma cells [67]. Treatment with UDCA in rats with experimental alcoholic liver disease (ALD) protected them from hepatosteatosis and liver damage [68]. The proposed mechanisms by which UDCA and TUDCA protect the liver from ethanol-induced damage include mitochondrial protection, improved ATP synthesis, and antioxidant activity against ethanol-induced reactive oxygen species (ROS) [69,70,71]. NorUDCA ameliorates experimental ALD, protects against hepatic inflammation, and activates hepatic peroxisome proliferator-activated receptor (PPAR)-γ [72]. UDCA was reported to increase the levels of protective prostaglandins that were depleted by alcohol treatment [73]. UDCA-Zein nanoparticles were shown to attenuate acute ALD in mice through antioxidant and anti-inflammatory mechanisms [74]. A clinical trial indicated that UDCA treatment attenuated bilirubin, gamma-glutamyl transpeptidase (GGT), and alanine aminotransferase (ALT) levels [75]. In another clinical study, 13–15 mg/kg/d of UDCA administration for six months attenuated serum GGT and alkaline phosphatase activity compared with placebo-treated individuals [76]. However, these improvements were accompanied by increased complications, decompensated liver cirrhosis, severe alcoholic cirrhosis, and jaundice, which required caution and decreased survival rates. An inappropriate dosage of UDCA could not be excluded as an explanation for the lack of therapeutic benefit [77,78]. There is scarce information on the utility of UDCA for ALD; therefore, no recommendation can be made yet (Figure 1).

### 2.3. Drug-Induced Liver Injury

Treatment of DILI is frequently limited to discontinuation of the drug causing liver damage and supportive therapy [4]. However, UDCA has been utilized to treat this condition alone or in combination with corticosteroids [79]. In particular, UDCA has been used to treat DILI caused by amoxicillin/clavulanate, flucloxacillin, bosentan, flutamide, tacrine, and anabolic steroids [80]. However, most studies have lacked a control group; therefore, a valid conclusion cannot be easily reached.

Several beneficial properties of UDCA for liver support make it useful in preventing DILI. UDCA has significant anticholestatic, antioxidant, anti-inflammatory, and antiapoptotic effects [81,82] (Figure 1).

#### 2.3.1. Antioxidant Activity of UDCA

DILI involves elevated ROS production [83,84,85,86]. In addition to the ability of UDCA to scavenge ROS [87], it has the capacity to trigger the nuclear factor-E2-related factor-2 (Nrf2) pathway [88,89,90,91], which upregulates antioxidant and cytoprotective molecules, including metallothionein, reduced glutathione (GSH), glutamate-cysteine ligase, and N-acetyl-L-cysteine [92,93], leading to a potent antioxidant and protective response. Therefore, UDCA’s strong antioxidant activity may be a useful tool for preventing and treating DILI.

#### 2.3.2. Anti-Inflammatory Activity of UDCA

Several anti-inflammatory mechanisms have been shown for UDCA. The translocation of UDCA to the nucleus activates the glucocorticoid receptor (GR), inducing an anti-inflammatory response [94,95]. In addition, UDCA downregulates activator protein-1 (AP-1) [96] to block the nuclear factor-κB (NF-κB) pathway [88]. Accordingly, UDCA inhibited mitogen-activated protein kinases and thus AP-1 activation, inhibiting the NF-κB pathway in RAW 264.7 macrophages challenged with lipopolysaccharide (LPS) [89].

#### 2.3.3. Antiapoptotic Activity of UDCA

Frequently, DILI causes liver damage by inducing apoptosis. In this context, UDCA likely inhibits mitochondrial permeability transition pores [90,91] via antioxidant mechanisms [97] and prevents apoptosis. In addition, UDCA can block apoptosis by translocating to the nucleus to bind GR, thereby inhibiting E2F-1 and p53-dependent Bax activation [98]. Moreover, tauroursodeoxycholate (TUDCA), an important UDC metabolite, inhibits Bax binding to mitochondria, thereby inhibiting apoptosis [99]. UDCA binds to the epidermal growth factor receptor to inactivate Bad, blocking apoptosis [100]. UDCA inhibits Janus kinase (JNK) activation by MAPK kinases to prevent bile acid-induced apoptosis [101]. Amaral et al. reported that UDCA decreased p53 DNA-binding activity and induced p53 degradation, thereby reducing apoptosis [102,103]. Zhang et al. reported that TUDCA binds to Bax [104] to block the Bcl-2-interacting domain (Bid)-dependent translocation of Bax to mitochondria, leading to the inhibition of apoptosis [99]. UDCA and TUDCA reduce bile acid-induced apoptosis by regulating AP-1 [96]. Moreover, UDCA decreased Fas ligand-induced apoptosis in mouse hepatocytes [105].

Furthermore, while endoplasmic reticulum (ER) stress can lead to apoptosis [106], TUDCA can attenuate ER stress, highlighting another antiapoptotic mechanism of this bile acid [107,108,109]; accordingly, UDCA reduces ER stress, decreasing caspase-12 activation [110].

Although several beneficial properties of UDCA suggest that this bile acid may help treat DILI, direct evidence of its utility remains scarce.

### 2.4. Cholestatic Liver Diseases

Cholestasis is a decrease in bile flow caused by any pathophysiological state. Chronic cholestasis can lead to fibrosis, cirrhosis, hepatic failure, and HCC [111]. The two primary forms of chronic cholestasis are PBC [112] and primary sclerosing cholangitis (PSC) [113]. PBC is characterized by chronic disruption of small and mid-sized intrahepatic biliary ducts, primarily affecting middle-aged women [114]. PSC is characterized by chronic destruction of the intra- and extrahepatic bile ducts, leading to inflammation, fibrosis, and cirrhosis [115]. The oral administration of UDCA, a hydrophilic bile salt, increases the hydrophilicity of the bile acid pool and decreases hydrophobic bile acid toxicity [116] and increases choleresis [117,118]. Other mechanisms have been described for the hepatoprotective effects of UDCA in biliary diseases, such as its ability to act as a Ca^2+^ agonist, an integrin and MAPK activator, and an inducer of vesicular exocytosis, thereby exerting a choleretic effect [111]. Moreover, according to “biliary HCO_3_^−^ umbrella” theory, HCO_3_^−^ produces an alkaline pH, attenuating hydrophobic bile acid permeation [119] (Figure 1).

Preclinical research has revealed significant beneficial effects of UDCA against cholestatic liver pathologies. UDCA reversed cholangiopathy and biliary fibrosis in Cyp2c70-deficient mice [112], and this drug prevented cholestasis in neonatal Cyp2c70-deficient mice [120]. NorUDCA exerts important choleretic activity independent of active enterohepatic cycling in rodents [112]. In addition to its anticholestatic properties, norUDCA also exerts anti-inflammatory effects by decreasing the T_H_17 cell-driven liver immune response [121,122].

The administration of UDCA to patients with PBC had significant positive effects on several markers of liver damage and pruritus [123]. Accordingly, placebo-controlled clinical studies have indicated that UDCA improved hepatic histopathological alterations and attenuated the progression of PBC to cirrhosis [124,125,126]. Therefore, given the lack of significant adverse effects, the European Association for the Study of the Liver recommends UDCA for patients with PBC [126].

However, the results of UDCA treatment in patients with PSC are less favorable than in patients with PBC. In a meta-analysis, Shi et al. reported that UDCA, despite improving serum markers of liver damage, had no beneficial effects on hepatic histology or survival in treated patients compared with the placebo group [127]. Similarly, a meta-analysis by Triantos et al. showed no impact on mortality, risk of cholangiocarcinoma, fatigue, pruritus, or disease progression [128]. Moreover, a study with high UDCA doses (17–23 mg/kg/d) did not reveal improvements in markers of liver injury, mortality, or need for liver transplantation [129]. Indeed, elevated UDCA doses are associated with several severe side effects [130]. Prolonged use of high doses of UDCA (28–30 mg/kg/d) has been observed to increase the development of esophageal varices in patients with early-stage PSC [131]. Furthermore, in PSC with inflammatory bowel disease treated with UDCA, most colonic carcinomas develop in the first years after the start of treatment [132].

In conclusion, UDCA and its derivatives are useful for the treatment of cholestatic liver diseases, MASH, ALD, and DILI. These bile salts protect against bile acid toxicity, oxidative stress, ER stress, cholestasis, inflammation, hepatosteatosis, apoptosis, and fibrosis by modulating several signaling pathways involved in these processes. UDCA is the only drug approved by the U.S. Food and Drug Administration for the treatment of PBC.

## 3. Pirfenidone

Pirfenidone, 5-methyl-1-phenyl-2[1H]-pyridone, is a valuable drug for treating lung fibrosis and has been approved by the FDA for the treatment of idiopathic pulmonary fibrosis [133,134,135,136,137,138,139]. Pirfenidone is known for its antifibrotic properties, and its mechanism of action involves attenuating signaling pathways related to scar tissue production, including the Wnt/glycogen synthase kinase (GSK)-3β/β-catenin and TGF-β1/Smad2/3 pathways in the lungs [136]. Pirfenidone has recently demonstrated diverse therapeutic potential in cholestatic liver injury in mice. The beneficial effects were associated with its dose-dependent inhibition of Wnt/GSK-3β/β-catenin/cyclin D1 signaling. The inhibition of β-catenin induces FXR and decreases bile acid deposition in the hepatic tissue [137]. Moreover, in primary rat intestinal fibroblasts, pirfenidone decreased the up-regulation of TGF-β1-induced collagen I and α-SMA by suppressing TGF-β1/Smad/CTGF signaling pathway [140]. In addition, the anti-inflammatory effects of pirfenidone are well documented. Tang et al. reported that pirfenidone attenuated inflammation by inhibiting macrophage polarization and the JAK2/STAT3 signaling pathway in a pulmonary rat silicosis model [137]. Pirfenidone mitigated inflammation and fibrosis by reducing interleukin (IL)-17A levels in a silica-induced pulmonary inflammation model in mice [141]. Notably, pirfenidone has been reported to exert its anti-inflammatory effects by inhibiting nucleotide-binding oligomerization domain-like receptor 3 (NLRP3) inflammasome signaling, as demonstrated in an LPS-induced lung inflammation model [139]. Pirfenidone increased IL-10 levels, an anti-inflammatory cytokine, thereby improving pancreatitis in murine models [141]. Pirfenidone markedly reduced serum ALT levels and suppressed NF-κB activation and TNF-α and IL-6 production [142].

**Figure 2:**
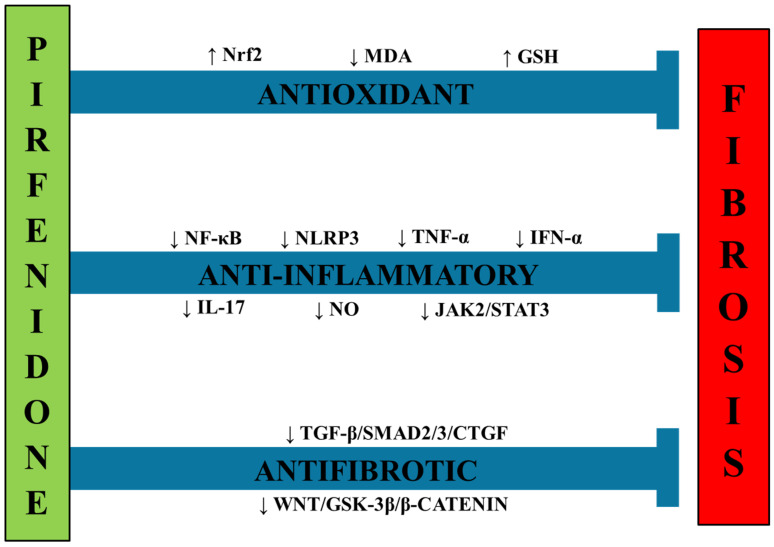
Pirfenidone exerts antifibrotic effects by downregulating oxidant, inflammatory, and fibrogenic pathways. Abbreviations: Nrf2: nuclear factor-E2-related factor-2; MDA: malondialdehyde; GSH: reduced glutathione; NF-κB: nuclear factor-κB; NLRP3: Nucleotide-binding oligomerization domain-like receptor family pyrin domain-containing 3; TNF-α: tumor necrosis factor-α; IFN: interferon; IL-17: interleukin-17; NO: nitric oxide; JAK2/STAT3: janus kinase 2/signal transducer and activator of transcription 3; TGF-β: transforming growth factor-β; SMAD2/3: suppressor of mother against decapentaplegic; CTGF: connective tissue growth factor; GSK-3: glycogen synthase kinase-3.

Regarding the antioxidant properties of pirfenidone, evidence indicates that this drug upregulates the Nrf2 signaling pathway, thereby activating the cell’s endogenous antioxidant machinery. Xie et al. reported that pirfenidone increased Nrf2 expression, which was dramatically reduced in mice fed a high-fat diet (HFD) to induce MASH; this effect was accompanied by normalization of oxidative stress markers, including malondialdehyde (MDA) levels and elevated GSH and SOD activities [143]. Other studies reported that pirfenidone increased the activity of the Nrf2 pathway in different organs, such as the lungs, in bleomycin-induced pulmonary fibrosis in mice [144] (Figure 2).

### Antifibrotic Properties of Pirfenidone in the Liver

While reports on the antifibrotic activity of pirfenidone in pulmonary idiopathic fibrosis are abundant, studies on its effects on liver fibrosis are scarce. Two clinical studies reported that pirfenidone administration to patients with fibrosis can improve fibrosis and patient outcomes [145,146,147]. Pirfenidone at a dose of 1200 mg significantly decreased non-invasive markers of liver fibrosis at 24 months [144]. These results may be explained by the ability of pirfenidone to inhibit the proinflammatory NF-κB signaling pathway [148], reduce TNF-α and IFN-α levels, and inhibit iNOS/NO induction [149]. Interestingly, pirfenidone downregulated key profibrogenic mediators, including transforming growth factor-β (TGF-β) and tissue inhibitors of metalloproteinase-1 and -2, thereby reducing scar tissue formation in a CCl_4_-induced fibrosis model in rats [150,151]. In another study, pirfenidone improved the Child–Pugh score in patients with chronic hepatitis C virus infection [146].

The reported beneficial effects of pirfenidone in patients with liver diseases are promising. Studies have shown that this multipotent pyridone analogue could inhibit fibrosis by negatively regulating the expression of diverse fibrogenic factors, inhibiting the production and release of inflammatory cytokines, and reducing cell damage from oxidative stress. A review of Ramírez-Mejía et al. showed that, in clinical trials, the large prospective cohort PROMETEO, the randomized controlled trial ODISEA, and the translational trial MINERVA, pirfenidone improved noninvasive markers of fibrosis, liver function tests, and quality of life [152]. These data suggest that pirfenidone may contribute to histological regression in advanced disease [152]. Basic and clinical researchers are encouraged to provide additional proof of the utility of this drug in liver diseases, as well as the mechanisms of action involved.

## 4. S-Adenosyl-L-methionine

S-Adenosyl-L-methionine (SAM) was discovered in 1951 and is the methyl donor utilized by methyltransferases for most transmethylation reactions in the body [153]. SAM plays an essential role in liver physiology and pathophysiology [154]. SAM is the product of methionine and ATP reactions catalyzed by methionine adenosyl transferases [155]. Once SAM donates a methyl group, it is converted to S-adenosylhomocysteine, a potent inhibitor of transmethylation, which is then converted to homocysteine (HCY). Through the activity of a synthetase, HCY can then form cystathionine, a precursor of GSH, or be methylated to produce methionine [156].

### S-Adenosyl-L-methionine Treatment in Liver Disease

SAM exhibits a significant first-pass effect when administered orally, resulting in poor bioavailability [157]. The liver half-life of SAM in healthy individuals is approximately 5 min [158]. New strategies are being implemented to improve drug bioavailability. A novel oral formulation of S-adenosylmethionine (MSI-195) has been developed in highly bioavailable enteric-coated tablets. The relative bioavailability of MSI-195 was approximately 2.8-fold that of SAM, thereby increasing MSI-195’sc bioavailability [157,158]. Furthermore, it was observed that the phytate anion protects SAM from degradation, likely due to steric hindrance, and that SAM phytate exhibited significantly better pharmacokinetic parameters than SAM itself when analyzed in an in vivo model. These results suggest the potential to use new SAM salts with improved pharmacokinetic properties [158].

Accordingly, Lu et al. [159] found that the level of SAM after an intraperitoneal administration peaked at 15 min and returned to baseline within 4 h. SAM has been evaluated in methionine- and choline-deficient MASH models and has been shown to attenuate disease progression [160] and prevent CCl_4_-induced liver fibrosis in rats [161]. The administration of SAM to rats with CCl_4_-induced cirrhosis protected against damage and alterations in the plasma membrane composition and function [162]. In the chronic bile duct ligation-induced cholestasis model, SAM treatment prevented liver damage and fibrosis and maintained the ultrastructure of the hepatic parenchyma [163]. Moreover, SAM exerted several beneficial effects on the livers of baboons chronically treated with alcohol [164]. SAM was demonstrated to prevent experimental HCC in rodents but was unable to confer a beneficial effect on established HCC [165]. Ansorena et al. [166] found that SAM can be proapoptotic in liver cancer cells but antiapoptotic in normal hepatocytes. SAM synthesis is decreased in MASH [167], cirrhosis [168,169], and HCC [170] and is correlated with a poor prognosis [171]. Treatment with SAM increases the GSH content in the livers of patients with liver disease [172] and survival in patients with alcoholic liver cirrhosis [173].

## 5. N-Acetylcysteine

N-acetylcysteine (NAC) is a precursor to L-cysteine and GSH and has been used as a mucolytic agent and an antidote for paracetamol-induced hepatotoxicity [174]. NAC, because of its antioxidant properties, has demonstrated multiple clinical applications, including the treatment of cancer, nephropathy, heart disease, pulmonary fibrosis, polycystic ovary syndrome, and influenza [175,176].

### 5.1. Drug-Induced Liver Injury

The generalized use of drugs in pharmacology has increased the number of cases of drug-induced liver injury (DILI) [177]. Notably, DILI is the principal determining cause of drug restriction or withdrawal from the market [178]. Because of its anatomical location between the gastrointestinal tract and the general circulation, the liver plays a fundamental role in the absorption and elimination of drugs. An overdose of paracetamol, also known as acetaminophen, is the most common cause of DILI because it is metabolized to N-acetyl-p-benzoquinone imine (NAPQI). This reactive product induces oxidative stress and covalently binds to sulfhydryl groups on peptides and proteins, disrupting their function [179]. NAPQI avidly binds to GSH, which serves to detoxify this metabolite; however, when an overdose of paracetamol is administered, the excess NAPQI exceeds GSH’s capacity to eliminate it, leading to oxidative stress as GSH is depleted [180]. Free radicals play pivotal roles in the initiation and progression of DILI [1,3,179,180,181]; therefore, antioxidants can be useful in treating this condition [181]. In this context, NAC can increase GSH levels, thus acting as an antioxidant and protective agent against NAPQI-induced toxicity [177] and other diseases [182,183,184]. Similarly, Yip and Dart reported that the administration of NAC 20 h after paracetamol intoxication effectively prevented liver damage [185]. Accordingly, Heard et al. reported that NAC attenuated hepatic injury caused by acetaminophen overdose if it was administered within 24 h of intoxication [186]. Another study showed that NAC prevented GSH depletion and did not interfere with the analgesic properties of acetaminophen in healthy patients [187]. Other reports have shown that NAC, at several doses, has a protective effect against paracetamol-induced liver damage [188,189]. Additionally, NAC administration protected against acetaminophen-induced toxicity in combination with other hepatoprotectors, such as calmangafodipir, a superoxide dismutase mimetic [190], or ondansetron [191].

### 5.2. Hepatic Fibrosis

Liver fibrosis is characterized by an abundance of extracellular matrix (ECM) proteins within the hepatic parenchyma, where oxidative stress and inflammation play crucial roles [1,2,3,192]. In addition to ROS, many cytokines and factors play important roles in fibrogenesis; among them, TGF-β is considered the most potent profibrogenic factor, activating HSCs to trigger fibrogenesis [193]. Galicia-Moreno et al. reported that NAC administration to rats treated with CCl_4_ prevented necrosis, cholestasis, and fibrosis and maintained the typical architecture of the hepatic parenchyma; these protective effects were attributed to the prevention of lipid peroxidation, preservation of hepatic GSH, and abrogation of TGF-β signaling increased by chronic CCl_4_ intoxication in the rats [194]. Moreover, NAC was investigated in a bile duct ligation (BDL) model of cholestasis in rats, and it was found that this compound effectively attenuated cholestatic markers, decreasing the serum markers of hepatic injury and exhibiting a potent antioxidant effect; moreover, NAC treatment prevented ECM deposition, inhibited TGF-β and IL-6 signaling, and increased the levels of the anti-inflammatory cytokine IL-10 in BDL rats [194]. These antifibrotic effects of NAC were subsequently corroborated by Nissar et al. in rats treated with CCl_4_ and thioacetamide [195].

**Figure 3:**
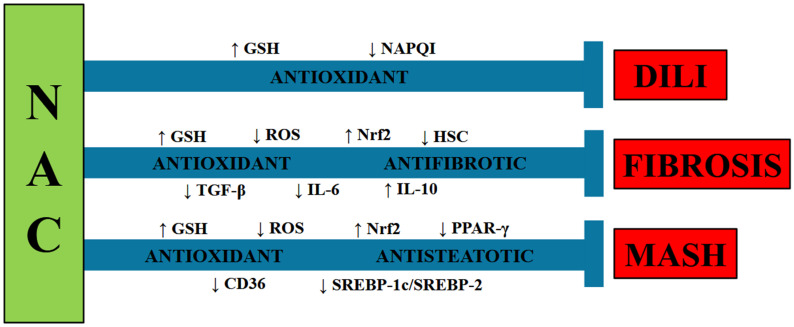
N-acetylcysteine (NAC) mitigates drug-induced liver injury (DILI), fibrosis, and metabolic dysfunction-associated steatohepatitis (MASH). Abbreviations: GSH: glutathione; NAPQI: N-acetyl-p-benzoquinone imine; ROS: reactive oxygen species; Nrf2: nuclear factor-E2-related factor-2; HSC: hepatic stellate cell; TGF-β: transforming growth factor-β; IL: interleukin; PPAR-γ: peroxisome proliferator-activated receptor-γ; SREBP: sterol regulatory element-binding protein.

In addition to the ability of NAC to serve as a GSH precursor, it also exerts antioxidant and cytoprotective effects by increasing the activity of the Nrf2 signaling pathway [196]. Considering that ROS can trigger HSC activation to induce fibrogenesis and that NAC exerts antioxidant effects by increasing the activity of the GSH and Nrf2 pathways, the antifibrotic properties of NAC can be attributed, at least in part, to its antioxidant activity (Figure 3).

### 5.3. Metabolic Dysfunction-Associated Steatohepatitis

#### 5.3.1. Antisteatotic Properties of NAC

Administration of NAC in drinking water (1 g/L) effectively reduced fatty liver, fatty acid synthesis, and plasma triglyceride levels in HFD-fed mice [197]. Accordingly, NAC administration reversed serum markers of liver injury, fatty liver, and other histopathological manifestations of MASH in rats [198]. Similarly, compared with untreated rats, MASLD rats coadministered metformin and NAC showed improved markers of liver damage and attenuated fatty liver [199]. Mechanistically, a report indicated that NAC could attenuate lipid deposition within the hepatic parenchyma by downregulating sterol regulatory element-binding protein (SREBP)-1c/SREBP-2 signaling [200]; others have shown that NAC exerts antisteatotic effects by decreasing cluster of differentiation 36 (CD36) [201] and PPAR-γ, and triggering the endogenous antioxidant system of the cell [202] in experimental models of MASLD.

#### 5.3.2. NAC Improves Hepatic Function by Antioxidant and Anti-Inflammatory Mechanisms

Samuhasaneeto et al. reported that NAC did not improve oxidative stress or fatty liver in a rodent model of MASLD [198]. However, the administration of NAC restored the disturbed lipid profile and improved hepatic steatosis in a mouse model of fatty liver induced by a high-cholesterol diet [203]. Accordingly, Baumgardner et al. reported that NAC ameliorated hepatic pathology in a rat model of MASH [204]. Tsai et al. demonstrated that long-term NAC treatment attenuated hepatosteatosis by reducing endoplasmic reticulum stress and the unfolded protein response in mice [205]. Research on the treatment of rats with MASH induced by a methionine and choline-deficient diet (MCD) with NAC or its derivatives has demonstrated that these compounds exert important antioxidant, antisteatotic and anti-inflammatory effects to attenuate/prevent this pathology [206,207,208,209,210,211]. Another study reported that NAC improved liver regeneration in rats with MASH, accompanied by antioxidant effects, as measured by increased GSH levels and attenuation of lipid peroxidation markers [212]. Using the transgenic ob/ob model of MASH, Laurent et al. demonstrated that NAC decreased lipid peroxidation and maintained mitochondrial GSH levels, alleviating the disease [213]. Several studies have demonstrated the antioxidant, antisteatotic, and anti-inflammatory effects of NAC in an ob/ob model of MASLD, improving mitochondrial function and oxidative phosphorylation [214,215,216,217].

#### 5.3.3. Clinical Evidence of the Effects of NAC on MAFLD in Patients

Few reports have been published on the impact of NAC on MASLD in patients. One study demonstrated that NAC reduced serum levels of liver damage markers, including ALT, AST, and gamma-glutamyl transferase [218]. Another report on children with parenteral nutrition-induced liver damage revealed that intravenous NAC administration improved erythrocyte GSH levels [219]. Metformin combined with NAC resulted in attenuation of both hepatosteatosis and MASH scores in patients [220]. Incubation with NAC improved the viability of human hepatocytes isolated from severely steatotic donor liver tissue [221]. In addition, NAC improved the liver function parameters of patients with MASH [222].

In summary, NAC has demonstrated important beneficial effects on various hepatic illnesses, including antioxidant, anti-inflammatory, antisteatotic, and antifibrotic effects (Figure 3). Given its safety profile and low toxicity, NAC can be used to treat patients with liver diseases; however, there is not enough evidence of its utility since more clinical trials are needed to determine the effectiveness of this compound.

## 6. Conclusions

Several drugs, including UDCA, pirfenidone, SAM, and NAC, have shown beneficial effects on a variety of liver diseases. These compounds have been tested in animal and cell models of liver injury; however, evidence of their utility in clinical trials has been limited. UDCA is an FDA-approved drug for the treatment of PBC. It has also been shown to have positive effects on other liver diseases, such as MASH, ALD, and DILI, via antioxidant and anti-inflammatory mechanisms, as well as protecting against toxic bile acids. Pirfenidone, used mainly as an antifibrotic agent in lung fibrosis, also exerts antifibrotic effects in the liver by modulating several pathways, including the Wnt/GSK-3β/β-catenin and TGF-β1/Smad2/3 signaling pathways. In addition, pirfenidone downregulates the proinflammatory NLRP3 inflammasome and NF-κB pathways and activates the antioxidant Nrf2 pathway in various organs and disease models. SAM has been proven to effectively prevent chronic liver damage, including fibrosis, in experimental models; however, no conclusive clinical reports indicate its utility in treating human fibrosis/cirrhosis/HCC. NAC is beneficial for the treatment of DILI, particularly acetaminophen intoxication, when it is administered at the proper time. Other beneficial effects on the liver, including the prevention or alleviation of hepatosteatosis, fibrosis, and cirrhosis, have been reported in experimental models. While these drugs are up-and-coming, much more fundamental and clinical research is needed before they can be recommended for treating human liver illnesses.

## 7. Limitations and Future Directions

The increasing prevalence of MASLD and its complications presents a significant challenge for pharmacology. Because the pathophysiology of liver diseases, such as ALD, is not fully understood, eliminating the causative agent does not, in some cases, halt the development of complications such as cirrhosis. Furthermore, some complications of liver disease led to dysfunction of other organs, such as the brain, kidneys, lungs, and cardiovascular system. Research is progressing in the development of agents targeting key inflammatory, metabolic, and fibrotic pathways, including GLP-1 and PPAR agonists. However, despite the recent FDA approval of resmetirom, few therapies are currently approved for MASH. Therefore, ursodeoxycholic acid, pirfenidone, S-adenosyl-L-methionine, and N-acetylcysteine could represent additional therapeutic strategies for the treatment of liver pathologies.

## Data Availability

No new data were created or analyzed in this study. Data sharing is not applicable to this article.
